# The Impact of Environmental and Social Responsibility on Customer Loyalty: A Multigroup Analysis among Generations X and Y

**DOI:** 10.3390/ijerph17186466

**Published:** 2020-09-05

**Authors:** Ovidiu-Ioan Moisescu, Oana-Adriana Gică

**Affiliations:** 1 Faculty of Economics and Business Administration, Babeș-Bolyai University, 400591 Cluj-Napoca, Romania; 2 Faculty of Business, Babeș-Bolyai University, 400038 Cluj-Napoca, Romania; oana.gica@tbs.ubbcluj.ro

**Keywords:** corporate environmental responsibility, corporate social responsibility, customer loyalty, customer–company identification, customer satisfaction, generation X, generation Y

## Abstract

The purpose of the current study was to comparatively estimate, for generation X and generation Y, the impact of corporate environmental and social responsibility on customer loyalty, via customer–company identification and customer satisfaction as mediators. For this, a survey was conducted among a sample of telecom customers, comprising 445 members of generation X, and 601 of generation Y. Data were analyzed using partial least squares structural equation modeling and multigroup analysis. Results revealed that the impact of corporate environmental responsibility on customer loyalty is significantly higher within generation Y, while the social facet of corporate responsibility is more relevant for customers from generation X. The current study contributes to the literature by developing and testing, within multiple generational groups, a theoretical model which outlines the links between environmental and social responsibility and customer outcomes. As these relationships have been scarcely analyzed in the context of age cohort membership as a moderator, our study fills an important literature gap, emphasizing significant differences between generations X and Y. The findings have relevant implications for the effective communication of corporate environmental and social responsibility activities, providing important insights on how messages and communication channels should be adapted to generations X and Y as target audiences.

## 1. Introduction

During recent decades, corporate environmental and social responsibility have attracted the attention of scholars from various scientific fields [1], being often referred to under the umbrella of wider concepts such as corporate social responsibility (CSR) or corporate sustainability.

From an organization’s perspective, environmental and social responsibility can have several positive outcomes concerning organizational attractiveness to employees, customers, investors and other stakeholders. Thus, organizational engagement in environmental and social responsibility is linked to a number of valuable attitudinal and behavioral employee outcomes, evidence suggesting that organizational CSR performance enhances companies’ attractiveness for both prospective and current employees [2], leading to higher employee satisfaction and outcomes [3]. Being perceived as an environmentally and socially responsible company also leads to reduced financing costs [4], better financial performance [5] and, consequently, to higher organizational appeal for actual and potential investors. Moreover, it can increase customers’ identification with the company, their satisfaction with its products/services, as well as their loyalty to the company, these positive effects of customers’ perceptions being the most frequently emphasized in the literature [6,7]. Therefore, it is extremely important for companies to be socially and environmentally responsible and to be perceived as such [8].

Current market trends and increased competition in most industries make it necessary for businesses to develop new models of sustainable business management that must include aspects related to improving customer loyalty [9]. Increased customer loyalty, by retaining existing customers and fostering positive word of mouth, can further lead to better business performance and long-term profitability [10,11]. It is, therefore, essential for businesses to acknowledge the importance of customer loyalty and, consequently, to work on improving all aspects that have been proven to have a significant impact on customer loyalty. Among these aspects, previous research has shown that customers’ perceptions of corporate environmental and social responsibility play an important role [6,7].

The relationship between perceived environmental and social responsibility, on one hand, and customer–company identification, customer satisfaction and customer loyalty, on the other hand, has been extensively investigated in the literature.

Thus, Walsh and Bartikowski [12], as well as Cuesta-Valino et al. [9], found that customers’ perceptions of a company’s CSR positively affect their loyalty to that company, both directly and indirectly, via customer satisfaction. Lee et al. [13], as well as Deng and Xu [14], also proved that perceived CSR has a positive direct effect on customer loyalty, depicting an indirect effect as well, mediated by customer–company identification. Other researchers [15,16] showed that customers’ perceptions of a company’s CSR have a positive impact on both customer–company identification and customer satisfaction and, also on customer loyalty. Summarizing, previous research has outlined a significant impact of companies’ perceived responsibility towards the environment and society on customer loyalty, both directly and indirectly, via mediators such as customer–company identification and customer satisfaction.

However, the moderating role of age and/or age cohorts (generations) within this complex set of relationships is still mostly an uncharted research topic. Generational differences should be expected, as different generations have developed their personalities, habits and opinions in distinct contexts in what concerns history, technology, values, philosophies, etc., all which can influence how groups of people born in certain years think and react. Therefore, people from different generations are expected to engage in different ways with brands, as well as to have distinct attitudes and reactions to companies’ efforts to be environmentally and socially responsible.

Previous studies have suggested that consumers’ age might have an impact on their attitudes and behavior towards the so-called “green” products/services [17,18,19,20], but most research on the subject have yielded contradictory results [21]. The few previously conducted studies aimed at investigating the moderating role of age in the relationship between perceived CSR and customer loyalty (e.g., [22,23]) suggest that age can be a moderating variable of this relationship. However, these studies ignored the complexity of the relationship which involves various indirect effects and important mediators such as customer–company identification and customer satisfaction.

The purpose of the current study was to comparatively estimate, for generation X and generation Y, the impact of corporate environmental and social responsibility on customer loyalty, via customer–company identification and customer satisfaction, as mediators of this relationship.

The current study contributes to the environmental and social responsibility literature by developing and testing, within multiple generational groups, a theoretical model which explicitly outlines the potential links between environmental and social responsibility and customer outcomes such as customer–company identification, satisfaction and loyalty. As these relationships have been scarcely analyzed in the context of generation affiliation as a moderator, our study fills an important literature gap, emphasizing significant differences between generations X and Y in what concerns the impact of environmental and social responsibility on customer loyalty, as well as the mechanism behind this impact, which involves the mediating role of customer–company identification and customer satisfaction. Considering its expected positive affect on organizational attractiveness to customers, CSR activities have become more than business ethics requirements, being nowadays important ingredients of how companies communicate with their customers, significant resources being assigned to regularly disseminate CSR efforts [24,25], in an attempt to improve reputation and increase customer loyalty. However, if there are generational differences in what concerns customers’ attitudes and responses to companies’ efforts to be environmentally and socially responsible, then CSR communication should be done differently among distinct generations, with emphasis on those CSR aspects that are relevant for each generation. Under these considerations, the current paper has practical implications in what concerns the appropriate dissemination of CSR activities, by taking into account age and age cohorts as a basis for market segmentation.

Furthermore, this paper will firstly include the theoretical background of the research and the research hypotheses, with focus on the concepts of environmental and social responsibility, customer-company identification, customer satisfaction, customer loyalty and inter-generational heterogeneity. Afterwards, the paper will describe the methodology involved by the study, with details regarding data collection, samples and measurements. The following section of the paper is dedicated to presenting and discussing the research results. Finally, the paper ends with a set of conclusions, as well as research limitations and future research directions.

## 2. Theoretical Background

### 2.1. Corporate Environmental and Social Responsibility

The concepts of corporate environmental and social responsibility have generally been placed either under the umbrella of corporate social responsibility (CSR), or under that of corporate sustainability.

Despite the fact that both CSR and corporate sustainability represent mainstream literature topics, the consensus regarding their definition is far from universal, while confusion still exists when CSR or corporate sustainability need to be systematized [26,27,28,29].

With regard to defining CSR, the literature emphasizes two main approaches: the four-part systematization [30] and, respectively, the stakeholder-based framework [27]. The four-part systematization proposed by Carroll [30] implies that CSR encompasses the economic, legal, ethical and discretionary (philanthropic) expectations that society has of organizations at a given point in time. On the other hand, according to the stakeholder-based framework proposed by Freeman et al. [27], CSR encompasses companies’ responsibilities towards their stakeholders, among which some of the most important are shareholders, employees, customers, the environment and the society.

A similar perspective was initially adopted by the European Commission (EC) who defined CSR in 2001 as “the voluntarily integration of social and environmental concerns in business operations as well as in interaction with stakeholders” [31]. As can be seen, the EC primarily emphasized social and environmental responsibilities within the larger concept of CSR. Ten years later, in 2011, the EC changed its definition of CSR putting even more importance on environmental and social responsibilities, officially redefining CSR as “actions by companies over and above their legal obligations towards society and the environment” [32].

In what concerns corporate sustainability, its definition is generally rooted into the wider definition of sustainable development which implies “meeting the needs of the present without compromising the ability of future generations to meet their own needs” [33]. Consequently, corporate sustainability has been defined as a business approach that creates long-term shareholder value by taking advantage of opportunities and managing risks related to economic, environmental and social developments [34]. Corporate sustainability implies meeting the needs of current stakeholders, without compromising its ability to meet the needs of future stakeholders as well, emphasizing the integration of economic, environmental and social aspects in companies’ development [35]. Overall, corporate sustainability involves the inclusion of social and environmental concerns in business operations and in interactions with stakeholders [36].

Summarizing, it can be stated that environmental and social responsibility are at the core of CSR and corporate sustainability. Inherently, due to the progressive increase in environmental concerns at international levels, the notion of Environmental Corporate Social Responsibility (ECSR) has emerged as a spin-off from CSR [37].

### 2.2. Customer–Company Identification

The concept of customer–company identification derives from that of organizational identification which is a person’s perception of “oneness or belongingness” with an organization [38]. Customer–company identification implies that a customer of a company sees him/herself as having similar attributes that he or she believes define that company [39].

In a more profound manner, customer–company identification is “the primary psychological substrate for the kind of deep, committed, and meaningful relationships that marketers are increasingly seeking to build with their customers” [40]. According to social identification theory, an individual identifying with a social category will feel his/her self-esteem enhanced and will do anything possible to preserve the attractiveness of that social category.

From a business–customer relationship perspective, companies represent social categories with which customers can identify [40]. Thus, consumers care not only about their customer experience but also want to belong to a social group when purchasing products or services [16].

A company’s perceived CSR represents an important component of corporate associations. Previous research has established that corporate associations affect consumer attitudes and behavior [40,41]. Particularly, CSR tends to have a positive effect on consumers’ attitude and behavior towards the focal company, including customer–company identification [15]. This happens because CSR induces customers to develop a sense of connection with the company [16]. Previous research has actually suggested that the way customers perceive a company’s environmental and social responsibility influences their identification with that company [15,16,42,43,44]. Therefore, we posited the following research hypotheses:

**Hypothesis** **1** **(H1).** *Perceived environmental responsibility has a positive influence on customer–company identification.*

**Hypothesis** **2** **(H2).** *Perceived social responsibility has a positive influence on customer–company identification.*

### 2.3. Customer Satisfaction

Customer satisfaction is often defined as the difference between customers’ expectations about a product or service and the actual performance received. More specifically, Fornell [45] defines customer satisfaction as “the consumer’s response to the evaluation of the perceived discrepancy between prior expectations and actual performance of the product as perceived after its consumption”. The same author emphasizes, however, that customer satisfaction would be better defined in a cumulative manner, as the overall evaluation based on the purchase and consumption experience with a product or service. Within a similar approach, Bitner and Hubbert [46] define customer satisfaction as an overall measure of how happy or content customers are with a product or service, taking all possible antecedents into consideration.

A company’s perceived environmental and social performance can increase the perceived value of its products/services [15]. This happens because customers, in their assessments, take into account both the economic and noneconomic value of products/services. In this context, the perceived environmental and social performance of a company represents a part of the noneconomic value of its products/services. Consequently, positive CSR associations can add extra perceived benefits/utilities to consumers, such as consumer self-enhancement, self-esteem etc. [15]. Furthermore, a higher perceived value should lead to increased customer satisfaction. Previous findings have shown that a company’s perceived environmental and social responsibility can positively influence customer satisfaction [15,16,43,47,48,49,50]. Hence, we issued the following research hypotheses:

**Hypothesis** **3** **(H3).** *Perceived environmental responsibility has a positive influence on customer satisfaction.*

**Hypothesis** **4** **(H4).** *Perceived social responsibility has a positive influence on customer satisfaction.*

According to the expectation–disconfirmation theory of customer satisfaction, as already stated, customers are more satisfied if the product/service performance exceeds or at least confirms prior expectations. From this perspective, customer–company identification offers a favorable context for customers to assess a product/service performance in comparison to prior expectations [51]. Thus, if expectations are confirmed or exceeded, customers with stronger identification with the company will be more satisfied because this reinforces their psychological attachment with the company and, consequently, their self-esteem. On the other hand, if expectations are not delivered properly, customers with stronger identification with the company will be less unsatisfied because they need to preserve their psychological attachment with the company and preserve their self-esteem. In this logic, customers with stronger identification with a company should be more satisfied with that company’s products/services. Previous research has actually suggested that when customers identify themselves with a company, the feeling of connection to that organization helps them achieve a positive social identity and make them feel more satisfied with its products or services [15,16,52]. In consequence, we issued the following research hypothesis:

**Hypothesis** **5** **(H5).** *Customer–company identification has a positive influence on customer satisfaction.*

### 2.4. Customer Loyalty

Customer loyalty represents the biased behavioral response expressed over time by consumers with respect to one or more alternative brands out of a set of brands, as a result of psychological processes [53]. Customer loyalty can be systematized as comprising cognitive, affective, conative and behavioral dimensions, being a deeply held commitment to rebuy a preferred product/service consistently in the future, thereby causing repetitive same-brand or same brand-set purchasing, despite situational influences and marketing efforts having the potential to cause switching behavior [54]. Nonetheless, repeat purchase behavior alone does not make a customer loyal, true loyalty also implies a psychological commitment and a positive company/brand attitude [55], including positive word-of-mouth.

As consumers value companies’ efforts to be environmentally friendly and good corporate citizens, business behaviors which are perceived as environmentally and socially responsible can foster consumers’ commitment to companies, thus leading to stronger customer loyalty [13]. Previous studies conducted in various industries have actually shown that positive perceptions regarding a company’s environmental and social responsibility can lead to higher customer loyalty [11,43,44,47,56,57]. Consequently, we issued the following research hypotheses:

**Hypothesis** **6** **(H6).** *Perceived environmental responsibility has a positive influence on customer loyalty.*

**Hypothesis** **7** **(H7).** *Perceived social responsibility has a positive influence on customer loyalty.*

Extant literature suggests that customer–company identification and satisfaction make customers more psychologically attached to companies [28], further enhancing customer loyalty [43,58]. Previous research [13,14] has proven that customer–company identification plays a significant role in improving customer loyalty, both directly and indirectly, via customer satisfaction. A strong identification with the company is likely expressible through a sustained, long-term preference for the company’s products or services, customer loyalty being thus a key consequence of customer–company identification [13]. Additionally, the general consensus in the literature is that customer satisfaction acts as an antecedent of customer loyalty [54], a satisfied client being more likely to repurchase the product/service and to recommend it to other potential customers via positive word of mouth [9]. Therefore, we posited the following two research hypotheses:

**Hypothesis** **8** **(H8).** *Customer–company identification has a positive influence on customer loyalty.*

**Hypothesis** **9** **(H9).** *Customer satisfaction has a positive influence on customer loyalty.*

### 2.5. Intergenerational Heterogeneity

A generation encompasses a series of consecutive birth years spanning roughly the length of time needed to become an adult [59], referring to a cohort of people born within a similar span of time, who not only share a comparable age and life stage, but were shaped by similar particular events, trends and developments [60].

As a consequence, members of a generation exhibit beliefs and behavior patterns that are different from those characterizing the members of other generations. These distinctions can refer to various contexts: as customers [59], as employees [61], as career seekers [62], etc.

Because different generational cohorts have different lifestyles and attitudes, this distinction is useful for understanding consumers’ decision process across age groups [63]. As different personal traits of younger and older consumers influence the way they relate to companies [64], perceptions and reactions to corporate environmental or social responsibility actions should be expected to contrast between different generations.

The moderating role of age and/or generation in the relationship between perceived environmental and social responsibility, on one hand, and customer loyalty, on the other hand, is mostly an uncharted research area.

Various studies have suggested that consumers’ age has a significant impact on their environmental knowledge, as well as on their attitudes and behavior towards the so-called “green” products/services [17]. Thus, previous research shows that younger generations are more concerned about environmental quality [18], buy “green” products more frequently than older ones [19], have a higher willingness to pay price-premiums for “green” products [65], appear to be more sensitive to environmental issues, more ecology-minded and more environmentally concerned than older generations [20,66,67], and have the education, motivation and social awareness to participate in the “green” movement [68]. However, most findings about the impact of consumers’ demographic characteristics such as age on their environmentally conscious behavior are contradictory [21].

With respect to companies’ social involvement, previous research has suggested that companies’ participation in the development of local communities, as well as their general contribution to societal advancement are more relevant to older individuals, both as consumers [64] and employees [69]. Moreover, older consumers tend to be more prone to socially responsible consumption and more sensitive to companies’ philanthropic activities than younger consumers, as once a stable social position is attained, older consumers tend to care less for their own benefits and become more concerned with the welfare of their communities [70].

The few previously conducted studies aimed at investigating the moderating role of age on the relationship between perceived CSR and customer loyalty (e.g., [22,23]) suggest that younger customers’ loyalty is impacted by companies’ perceived environmental responsibility to a larger extent than in the case of older customers, while companies’ perceived responsibility towards local communities has a relatively higher impact on older customers’ loyalty than in the case of younger customers. However, most of these studies did not take into account relevant mediators such as customer–company identification, customer satisfaction and others alike, considering only the moderating effect on the direct impact of perceptual CSR on customer loyalty.

Taking into account these arguments, we issued the following research hypotheses:

**Hypothesis** **10** **(H10).** *The positive influence of perceived environmental responsibility on customer–company identification, customer satisfaction and customer loyalty is higher within customers from generation Y than within those from generation X.*

**Hypothesis** **11** **(H11).** *The positive influence of perceived social responsibility on customer–company identification, customer satisfaction and customer loyalty is higher within customers from generation X than within those from generation Y.*

## 3. Materials and Methods

Data were collected via a questionnaire-based survey conducted among customers of the four major telecom service providers in Romania: Orange, Vodafone, Telekom and Digi. We selected the telecom industry because we wanted to reach customers with a consistent knowledge of environmental and social responsibility activities and policies implemented by the industry’s main competitors. People use telecom services extensively, while at the same time the Romanian mass media cover the industry comprehensively [71]. Furthermore, consumers’ perceptions regarding the industry’s competitors are shaped by their direct experiences with such companies and by their exposure to mass media information. Additionally, the telecom sector, which can be described as an oligopoly with few large corporate competitors, regardless of the country or region taken into consideration, is generally involved in CSR actions, having robust CSR policies and procedures implemented, and paying particular attention to regularly and fully disclosing and communicating their CSR activities in structured reports, which are made publicly available via their own media and disseminated via paid media or publicity. Therefore, the telecom industry was considered a good option for our study.

The survey resulted in 1464 paper and pencil self-administered completed questionnaires. Straight-liners, as well as respondents who did not qualify to be considered among the X and Y generational groups were removed from the sample. As a result, the final validated sample analyzed in the current research comprised 1046 Romanian telecom customers.

Data collection was performed by a large team of business students who distributed and retrieved the questionnaires and who were afterwards rewarded for their contribution to the process. Students were recruited based on their residence so that the survey could reach various geographical regions.

The sampling procedure was nonprobabilistic, consisting of quota sampling by gender and age, capitalizing on a significant snowball sampling effect, given the large number of survey operators involved in data collection (more than one hundred bachelor and master students) and their personal acquaintances networks. In order to minimize sample selection bias, each survey operator was instructed to apply the questionnaire to a certain number of men and women, as well as to a specified number of respondents from several specified age group. The data were collected in 2016, before the adoption of the EU’s General Data Protection Regulation (GDPR).

Even though the conceptualization of generations X and Y is still under debate, considering the extant literature on intergenerational heterogeneity [59,60,72], participants in the study born between 1965 and 1980 were classified as generation X, while those born between 1981 and 1997 were classified as generation Y. Hence, the investigated sample comprised 445 members of generation X and 601 respondents from generation Y, each subsample being quasi-evenly split for gender and covering the full spectrum of birth years. Table 1 depicts the detailed demographics of the sample.

According to the data publicly available on the website of the Romanian National Institute of Statistics, in line with the previously specified generational thresholds, the population of Romania at the end of 2016 (the year of data collection) comprised 4.2 members of generation Y and 4.8 million of generation X. As the data collection procedure implied quota sampling by gender and age, our generation Y and X samples’ structure considering these demographics resembles to a reasonable extent the structure of the investigated generational populations, as can be seen in Table 2.

The measurements employed in this study were all based on scales previously developed and validated in the literature for assessing customer–company identification, customer satisfaction, customer loyalty and consumers’ perceptions of corporate social responsibility. Customers’ perceptions of corporate environmental responsibility were measured using 3 items, while perceived social responsibility was assessed using 4 items, all adapted from Öberseder et al. [44]. In order to measure customer–company identification, 3 items were adapted from Mael and Ashforth [73]. Customer satisfaction and customer loyalty were assessed using a set of 3 items for each, drawn from the scales developed by Cronin et al. [74] and, respectively, Zeithalm et al. [75].

All five latent variables employed in the current study were conceptualized as reflective (items are presented in detail in Table 3). This type of measurement was adopted for two reasons: firstly, the previously validated scales which we adopted in the current study were initially conceptualized as reflective by their developers, and, in order for the measurements to be valid, we needed to be in line with their original conceptualization; secondly, for each latent variable, items represent manifestations of the corresponding constructs (e.g., customer–company identification), indicator items associated with each particular construct being expected to be correlated with each other [76]. In the case of reflective latent variables, individual items are interchangeable, and any single item can generally be left out without changing the meaning of the construct and its validity, as long as the construct has sufficient reliability [76]. Therefore, the reduced number of items used in order to measure each construct does not represent a measurement validity issue, but rather prevents respondent fatigue and decreased response rates.

Participants in the study were firstly asked to state their current telecom service provider. Furthermore, all answers were given with reference to the specified company (e.g., “I believe that this company …”, “This company …” etc.). All items were measured on a Likert scale ranging from 1 = “strongly disagree” to 7 = “strongly agree”. We opted for the extended Likert scale (from 1 to 7) instead of the original one (from 1 to 5), as the psychometric literature suggests that having more scale points can provide deeper and more robust measurements, especially when attitude related variables are involved in the research, such as in our case [76].

**Figure 1:**
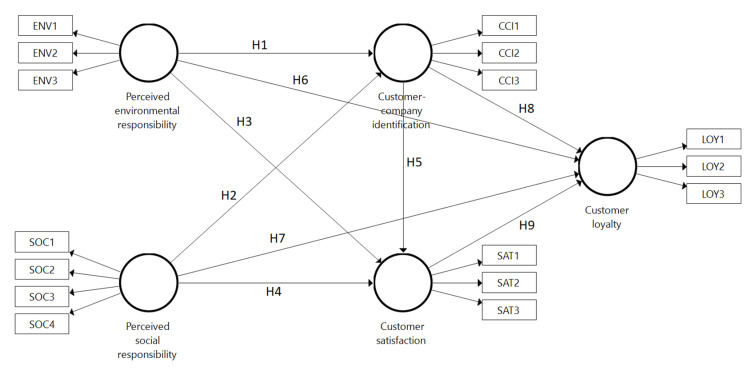
Research framework.

Research hypotheses were put together into a structural equation model (see Figure 1), which was further analyzed by means of partial least squares structural equation modeling (PLS-SEM).

The choice to use the PLS-SEM technique in order to assess the research model relationships was based on its capability to provide a balance between explanation and prediction [77]. Causal explanations were at the core of our research hypotheses. At the same time, being intended to comparatively estimate the impact of corporate environmental and social responsibility on customer loyalty, for generation X and generation Y, our model was expected to have predictive relevance and to yield meaningful managerial implications. Therefore, being focused on prediction, PLS-SEM was considered the best choice for data analysis. Furthermore, the SmartPLS 3 software (SmartPLS GmbH, Boenningstedt, Germany) was used to estimate the model parameters [78].

## 4. Results

The first stage of our PLS-SEM analyses consisted of assessing the measurements. As all measures were reflectively conceptualized, we evaluated our constructs’ internal consistency reliability, their convergent validity, as well as their indicators’ reliability. As can be seen in Table 4, all five latent variables exhibited very good internal consistency; composite reliability values were above the recommended threshold of 0.7 in all cases, without exceeding 0.95, as suggested by Hair et al. [76]. In what concerns convergent validity, the average variance extracted values were above 0.5 in all cases, indicating that our latent variables are convergent [76]. Regarding indicators’ reliability, outer loading values exceeded the threshold of 0.7 for all items, except for one included in the perceived social responsibility measurement scale, which had a loading of 0.677. Hair et al. [76] suggest that an indicator with an outer loading between 0.4 and 0.7 should be considered for removal only if deleting it significantly increases composite reliability or average variance extracted. However, this was not the case. Moreover, the outer loading of this indicator was extremely close to the cutoff value, and removing it would have led to lower content validity for the construct representing perceived social responsibility, as it referred to supporting charitable and social projects and facilities. Therefore, the item was kept in the scale.

Our model’s reflective measurements also needed to be assessed for discriminant validity, as constructs had to be distinct from each other in order for the structural model to be reliably assessed. We evaluated discriminant validity using both the Fornell–Larcker and the heterotrait–monotrait ratio of correlations (HTMT) criteria [79]. As can be seen in Table 5, in the case of the Fornell–Larcker criterion, the correlation between each pair of constructs (under diagonal) was lower than the square root of the average variance extracted for each construct (diagonal). This indicates discriminant validity. Nonetheless, recent research [71,80] has shown that the HTMT criterion is more conservative and it should be used in order to test whether constructs are truly distinct from each other. HTMT results in Table 5 also indicate discriminant validity, as HTMT values are positioned under the threshold of 0.85, as suggested by Henseler et al. [79]. There is, however, an exception, in the case of the loyalty–satisfaction pair of constructs, which yields a HTMT value of 0.875. Nevertheless, this is not a discriminant validity issue, as when constructs are conceptually similar (such as in the case of satisfaction and loyalty), Henseler et al. [79] suggest a cutoff value of 0.9.

Before comparing parameter estimates between generation X and Y, we needed to ensure measurement invariance. Otherwise, we could not be confident that group differences in model estimates did not result from the different content or meanings that latent variables might have had across age generations [81]. In order to assess measurement invariance, we employed the measurement invariance of composite models (MICOM) procedure developed by Henseler et al. [82]. As we needed to compare two groups, we assessed configural and compositional invariance. Given the fact that we used identical indicators, identical data treatment techniques and identical algorithm settings for both generation X and Y, configural invariance was automatically ensured. In what concerns compositional invariance, permutation testing results outlined in Table 6, more specifically the permutation *p*-values which are all considerably larger than 0.05, indicate that compositional invariance was established for all our five constructs. Therefore, comparing our model’s parameter estimates between generation X and Y is feasible.

As configural and compositional invariance between the two investigated customers groups was established for all variables, we further tested our proposed model comparatively, within customers from generations X and Y. In order to do this, we used nonparametric tests, as suggested by Hair et al. [81] for comparing two groups with PLS-SEM. More specifically, we ran the permutation test developed by Chin and Dibbern [83], with 1000 permutations, as well as the PLS-MGA procedure, developed by Henseler et al. [84], based on bootstrapping with 5000 subsamples. The results are presented in Table 7.

As can be seen, results show that there were significant positive direct effects of perceived environmental responsibility and perceived social responsibility on customer–company identification, within both generations. Therefore, Hypotheses H1 and H2 were confirmed. Additionally, this impact of perceived environmental responsibility is higher than the impact of perceived social responsibility, for both generations. However, it can be stated that there was no significant difference between generation X and generation Y with regard to the impact of environmental or social responsibility on customer–company identification, as both permutation and PLS-MGA *p*-values were consistently above the thresholds of 0.05 and 0.10. Consequently, hypotheses H10 and H11 were not confirmed for this relationship.

Results also show that there was a significant positive direct effect of perceived environmental responsibility on customer satisfaction within generation Y, but not within generation X. The difference between the two generations was significant in this case, as shown by both permutation and PLS-MGA *p*-values which were below the threshold of 0.05. Accordingly, Hypothesis H3 was only confirmed for generation Y, and H10 was confirmed in the case of this relationship.

Furthermore, results also indicate that there was a significant positive direct effect of perceived social responsibility on customer satisfaction within both generations; therefore, Hypothesis H4 was confirmed both for generation X and generation Y. However, permutation and PLS-MGA *p*-values (which were below the threshold of 0.05) suggest that there was a significant difference between generation X and generation Y in what concerns this relationship. More specifically, the impact of perceived social responsibility on customer satisfaction was significantly higher for generation X than for generation Y. Thus, Hypothesis H11 was confirmed for this relationship.

Perceived environmental and social responsibility also have indirect positive effects on customer satisfaction, the relationship being mediated by customer–company identification. Even though this indirect effect was rather low, it can be seen that it was higher in the case of environmental responsibility, compared to social responsibility. Nevertheless, there was no significant difference between generation X and generation Y in what concerns these indirect relationships (permutation and PLS-MGA *p*-values above 0.10).

With regard to Hypothesis H5, it can be stated that the direct impact of customer-company identification on customer satisfaction was confirmed as being positive and significant for both generations, with no significant difference between generation X and generation Y (permutation and PLS-MGA *p*-values above 0.10).

Furthermore, results show that there was no significant direct effect of perceived environmental responsibility on customer loyalty, neither within generation X, nor within generation Y. Therefore, Hypothesis H6 was rejected for both generations, as well as Hypothesis H10 in the case of this relationship (as there are no intergenerational differences in what concerns this direct relationship).

However, there was a significant positive indirect effect of perceived environmental responsibility on customer loyalty, mediated by customer–company identification and customer satisfaction; this indirect effect was different in size for generation X and generation Y, at a 10% level (permutation and PLS-MGA *p*-values below the threshold of 0.10). Thus, this indirect effect was higher within customers from generation Y than for customers from generation X. This result partially confirmed Hypothesis H10 for the relationship between perceived environmental responsibility and customer loyalty.

Results suggest that there was a significant positive direct effect of perceived social responsibility on customer loyalty within both generations; therefore, Hypothesis H7 was confirmed for both generation X and generation Y. Permutation and PLS-MGA *p*-values (which were above the threshold of 0.10) suggest that there was no significant difference between generation X and generation Y in what concerns this direct relationship, and, therefore, Hypothesis H11 was rejected in this case.

Nevertheless, there was a significant positive indirect effect of perceived social responsibility on customer loyalty, mediated by customer–company identification and customer satisfaction. This indirect effect was different in size for generation X and generation Y, as permutation and PLS-MGA *p*-values were below the threshold of 0.05. If path coefficients were scrutinized, it would be stated that this indirect effect was higher within customers from generation X than for customers from generation Y. This outcome partially confirmed Hypothesis H11 for the relationship between perceived social responsibility and customer loyalty.

With regard to hypotheses *H8* and *H9*, it can be stated that the direct impacts of customer-company identification and customer satisfaction on customer loyalty was confirmed as being positive and significant for both generations, with no significant difference between generation X and generation Y (permutation and PLS-MGA *p*-values above 0.10). As expected, customer satisfaction had a much higher direct impact on customer loyalty that customer–company identification.

Besides the direct impact, customer–company-identification also had an indirect impact on customer loyalty, via customer satisfaction; this relationship was similar among generation X and generation Y, with no significant differences between generations (permutation and PLS-MGA *p*-values above 0.10).

Overall, considering R square values, it can be stated that our proposed model explained 62.5% of the variance of customer loyalty for generation X, and 64.2% for generation Y. Consequently, the model has explanatory relevance. However, there was no significant difference between the two generations in what concerns the explanatory power of our model, as corresponding permutation and PLS-MGA *p*-values were way above the cutoff value for statistical significance.

As the purpose of the current study was to comparatively estimate, for generation X and generation Y, the impact of corporate environmental and social responsibility on customer loyalty, we further needed to assess our model’s predictive relevance for each of the two generational groups. Only with predictive relevance established, we could further outline meaningful and scientifically sound practical implications.

In order to assess the model’s predictive power, we employed the PLSPredict procedure, a holdout sample-based procedure that generates case-level predictions on endogenous item level [85,86]. Results are focused on the target endogenous variable—customer loyalty—and are outlined in Table 8.

We used 10 folds and the same number of replications, comparing the RMSE (root mean squared error) values from the PLS-SEM analysis with those generated by a naive linear model (LM) benchmark. Firstly, as all customer loyalty indicators yield Q2_predict values above zero, it can be stated that comparing RMSE for PLS-SEM and the LM benchmark is feasible. As RMSE values from the PLS-SEM analysis were lower than those generated by the LM benchmark for all indicators, both for generation X and generation Y, we can conclude that our model has high predictive power for both generations. Consequently, practical implications can be outlined and would be scientifically sound.

## 5. Discussion and Implications

The current study’s results indicating a positive and direct impact of perceived corporate environmental and social responsibility on customer loyalty are consistent with those previously obtained by Lee et al. (2012), as well as by Walsh and Bartikowski (2013). The fact that this direct impact is significantly lower compared to the indirect effect (mediated by customer–company identification and customer satisfaction) is also in line with the previous findings mentioned above.

The positive direct influence of perceived corporate environmental and social responsibility on customer–company identification confirms previous findings revealed by He and Li [15], Matute-Vallejo et al. [16], Lee et al. [13] and Deng and Xu [14]. Furthermore, the direct and positive effect of perceptual CSR on customer satisfaction is also consistent with the results previously obtained by He and Li [15], Matute-Vallejo et al. [16], Walsh and Bartikowski [12] and Cuesta-Valino et al. [9]. Our findings also indicate that the impact of perceived CSR on customer–company loyalty was lower than on customer satisfaction, just as He and Li [15] previously suggested in their research.

The novelty of the current study consists of the depiction of the moderating role of customers’ generational affiliation within this complex set of relationships, as this is still mostly an uncharted research topic. Thus, considering our main target variables (i.e., customer satisfaction and customer loyalty), perceived environmental responsibility was found to be significantly more relevant to customers from generation Y, than to customers from generation X. On the other hand, perceived social responsibility plays a more important role for generation X than for generation Y.

This can be explained by the distinct contexts in which the two investigated generations have set their long-term attitudes, personalities and values. Thus, the premises for the development of a public environmental consciousness appeared during the 1970s, when government environmental agencies and the UN environment program were created. However, this trend became prominent only later, during the 1980s and especially during the 1990s. The start of the 1990s also saw the fall of communism across Central and Eastern Europe, which further led to a growing public environmental consciousness in this region in the 1990s and 2000s. Therefore, Generation Y members, who have formed their personalities and established their values during the 1990s and 2000s should be expected to be more environmentally concerned than members of Generation X.

Additionally, as a recent report from the American Marketing Association [87] shows, members of generation Y look out for CSR before they buy, almost three quarters of millennials being willing to pay extra for products offered by environmentally responsible companies. In other words, for many members of generation Y, environmental sustainability is not just ”nice to have” but a primary reason to either buy or not buy from a company.

Considering the model’s explanatory relevance and predictive power, our research results have both theoretical and practical implications.

From a theoretical perspective, our results add to the extant knowledge regarding the relationship between customer loyalty and corporate environmental and social responsibility. The current study reinforces various previous findings that support the idea of a positive direct and indirect impact of perceived environmental and social responsibility on customer loyalty. Additionally, it confirms that customer–company identification and customer satisfaction are relevant mediators of this relationship. However, as this relationship has been scarcely analyzed in the context of moderators such as age or generation, our study fills an important literature gap, emphasizing significant differences between generations X and Y in what concerns the impact of environmental and social responsibility on customer loyalty, and the whole mechanism behind this impact (i.e., mediation by customer–company identification and customer satisfaction).

As for practical implications, our results firstly emphasize that in order to increase customer loyalty, companies need to engage in environmental and social responsibility activities, and to employ adequate strategies and policies regarding their impact on the environment and communities. Our research proves that being positively perceived as an environmentally and socially responsible company can lead to higher customer–company identification and customer satisfaction, and consequently to higher customer loyalty. Therefore, companies should work diligently to gain the status of responsibility in the eyes of their customers. This means that businesses not only need to be and do good in relation to the environment and local communities, but they also need to actively communicate this among their customers.

By engaging in environmental and social responsibility activities, companies can generate better support and advocacy behaviors among their customers. However, customers’ low awareness of such activities can compromise companies’ attempts to maximize business benefits from their CSR activities [88]. Therefore, it is extremely important that businesses communicate their environmental and social responsibility effectively. In order to do this, they need to have a deep understanding of the key issues related to CSR communication, and to adapt their CSR related messages and communication channels to specific target audiences. From this perspective, considering our results, generations X and Y should be addressed distinctively, with more emphasis on social responsibility activities when communicating to generation X, and more focus on environmental responsibility when generation Y is the communication target. Moreover, even though previous research has already shown that more environment-friendly companies can acquire higher market shares [89], our study suggests that this is applicable to a greater extent when targeting generation Y consumers.

Previous research has pointed out that companies can efficiently foster the business benefits of their environmental and social responsibility activities, only when the CSR issues to be communicated, the content of CSR related messages and the communication channels are selected/adjusted accordingly to specific market segments [6,90]. Based on our results, customer segments based on age and generations are essential in this context. Therefore, corporate environmental and social responsibility related messages and their channels of dissemination should be adapted to each generation-based target audience. For example, resent research has shown that different generations prefer specific communication channels for information about brands and companies [91]. Consequently, companies should prepare personalized CSR reports for customers from generation Y and X, and disseminate these reports via social media and email to generation Y, and via email and offline channels (e.g., mail, point of service/sales etc.) to generation X.

Taking into account our research context, we can state that telecom companies which operate in European developing countries can enhance their customer loyalty by actively communicating and disclosing their environmental and social policies and actions. However, this communication needs to be performed differently for generation X and generation Y. As our results show, even though customer loyalty is positively and significantly impacted (directly and indirectly) by both environmental and social responsibility, customers from generation Y tend to put more importance on environmental responsibility, while those from generation X consider social and community responsibility to be more relevant. Consequently, when communicating about their CSR activities, telecom companies should personalize their messages and reports according to the generation with which they are communicating. Thus, if they target customers from generation Y, they should emphasize aspects such as using environmentally friendly materials, being preoccupied with recycling and striving to minimize the consumption of resources. On the other hand, if they communicate to customers from generation X, companies should highlight aspects such as engaging in charitable and social projects; contributing to economic development; creating jobs in the region; and respecting regional values, customs and culture.

## 6. Conclusions

The current study’s results show that there is a positive influence of perceived corporate environmental and social responsibility on customer loyalty. However, the direct impact is rather low, the total effect being mainly indirect, mediated by customer–company identification and, especially, customer satisfaction.

Additionally, our results suggest that there are significant differences between customers from generation X and those from generation Y concerning this impact and the mechanism behind it.

More specifically, there is a significant positive direct effect of perceived environmental responsibility on customer satisfaction within generation Y, but not within generation X. Even though there is a significant positive direct effect of perceived social responsibility on customer satisfaction within both generations, this impact is significantly higher for generation X than for generation Y.

Regarding the indirect effects on customer loyalty, our research shows that there is significant positive indirect effect of perceived environmental responsibility on customer loyalty, mediated by customer–company identification and customer satisfaction, but this indirect effect is higher within customers from generation Y than for customers from generation X. On the other hand, the significant positive indirect effect of perceived social responsibility on customer loyalty is higher within customers from generation X than for customers from generation Y.

Overall, the current study emphasizes a higher focus on environmental responsibility within generation Y customers, and more importance put on social and community responsibility by customers from generation X.

From a practical perspective, we point out that in order to increase customer loyalty, companies should effectively communicate their environmental and social responsibility activities to their customers. Thus, businesses need to personalize their CSR related messages and to choose appropriate communication channels based on customers’ generational affiliation. More specifically, generations X and Y should be targeted distinctively, with more emphasis on social responsibility activities when communicating with generation X, and more focus on environmental responsibility when generation Y is the communication target.

This research has several limitations which, at the same time, represent opportunities for future research directions.

Firstly, the multigroup analysis takes into account only two generational groups—generation X and generation Y—omitting other generations. As a future research direction, the investigated relationships should be compared among generations X, Y and Z, the latter representing an important part of the customer-base for many businesses.

Secondly, the research is conducted within a specific geographical and cultural context (Romania: a developing country) and a specific industry (telecom). Therefore, our results can be extrapolated to other countries or industries only with certain limitations and precautions. Consequently, as a future research direction, the investigation should be extended internationally, taking into account several locations and cultures (i.e., customers from European and non-European countries, both developed and developing), other services industries (e.g., banking), as well as nonservice industries.

Thirdly, the current study focuses on environmental and social responsibility, omitting other facets of corporate sustainability that might have an impact on customer loyalty, such as, for example, corporate responsibility in relation to customers or employees. Especially considering the recent COVID-19 pandemic, customers’ perceptions related to whether companies ensure customer and employee safety might have become extremely important. Therefore, future research should encompass such aspects besides corporate environmental and social responsibility, in order to depict their influence on customer loyalty and the potential differences between generations with respect to this relationship.

## Figures and Tables

**Table 1:** Sample demographics.

Demographic	Generation Y (Born 1981–1997)	N	%	Generation X (Born 1965–1980)	N	%
**Gender**	Men	289	48.09	Men	213	47.87
	Women	312	51.91	Women	232	52.13
	**Total**	**601**	**100**	**Total**	**445**	**100**
**Year of birth**	1997	20	3.33	1980	22	4.94
	1996	29	4.83	1979	25	5.62
	1995	28	4.66	1978	30	6.74
	1994	44	7.32	1977	21	4.72
	1993	43	7.15	1976	25	5.62
	1992	38	6.32	1975	25	5.62
	1991	39	6.49	1974	26	5.84
	1990	53	8.82	1973	30	6.74
	1989	49	8.15	1972	29	6.52
	1988	52	8.65	1971	31	6.97
	1987	37	6.16	1970	30	6.74
	1986	21	3.49	1969	40	8.99
	1985	36	5.99	1968	41	9.21
	1984	29	4.83	1967	23	5.17
	1983	28	4.66	1966	19	4.27
	1982	33	5.49	1965	28	6.29
	1981	22	3.66			
	**Total**	**601**	**100**	**Total**	**445**	**100**
**Education**	HS−	267	44.43	HS−	248	55.73
	BA	206	34.28	BA	125	28.09
	MA+	128	21.29	MA+	72	16.18
	**Total**	**601**	**100**	**Total**	**445**	**100**

Note: HS− = High-school or lower education; BA = Bachelor studies; MA+ = Master studies or higher.

**Table 2:** Sample vs. population demographics.

		% in Gen Y Sample	% in Gen Y Population *		% in Gen X Sample	% in Gen X Population *
**Gender**	Men	48.09	51.72	Men	47.87	51.19
	Women	51.91	48.28	Women	52.13	48.81
	**Total**	**100**	**100**	**Total**	**100**	**100**
**Year of birth**	1981–1985	24.63	31.47	1965–1969	33.93	31.11
	1986–1991	41.76	37.96	1970–1974	32.81	31.01
	1992–1997	33.61	30.57	1975–1980	33.26	37.88
	**Total**	**100**	**100**	**Total**	**100**	**100**

* According to the Romanian National Institute of Statistics, http://statistici.insse.ro:8077/tempo-online/.

**Table 3:** Measurements.

Measurement	Code	Item
Perceived environmental responsibility	ENV1	Works diligently to use environmentally friendly materials
	ENV2	Is concerned with recycling and waste management
	ENV3	Strives to minimize the consumption of resources
Perceived social responsibility	SOC1	Supports charitable and social projects and facilities
	SOC2	Contributes to the economic development of the region
	SOC3	Creates jobs in the region
	SOC4	Respects regional values, customs, and culture
Customer-company identification	CCI1	I feel angry when someone criticizes this company
	CCI2	I feel good when someone praises this company
	CCI3	I am interested in what others think about this company
Customer satisfaction	SAT1	This company’s products/services are exactly what I need
	SAT2	My choice to become this company’s customer was a very good one
	SAT3	I am very satisfied with this company
Customer loyalty	LOY1	This company is my first choice in its sector
	LOY2	I would recommend this company to my friends/acquaintances
	LOY3	I will continue to be a customer of this company

**Table 4:** Indicator reliability, construct internal consistency reliability and construct convergent validity assessment.

Construct	Item	Outer Loadings	Composite Reliability	Average Variance Extracted (AVE)
Perceived environmental responsibility	ENV1	0.891	0.897	0.743
	ENV2	0.837		
	ENV3	0.858		
Perceived social responsibility	SOC1	0.677	0.810	0.517
	SOC2	0.753		
	SOC3	0.729		
	SOC4	0.714		
Customer-company identification	CCI1	0.914	0.893	0.737
	CCI2	0.888		
	CCI3	0.766		
Customer satisfaction	SAT1	0.913	0.940	0.840
	SAT2	0.919		
	SAT3	0.918		
Customer loyalty	LOY1	0.898	0.931	0.819
	LOY2	0.896		
	LOY3	0.921		

**Table 5:** Discriminant validity assessment.

Fornell–Larker Criterion						Heterotrait–Monotrait (HTMT) Ratio of Correlations				
	LOY	SAT	CCI	ENV	SOC		LOY	SAT	CCI	ENV
LOY	0.905					LOY				
SAT	0.788	0.917				SAT	0.875			
CCI	0.368	0.356	0.858			CCI	0.419	0.404		
ENV	0.315	0.347	0.384	0.862		ENV	0.366	0.401	0.463	
SOC	0.353	0.351	0.298	0.480	0.719	SOC	0.450	0.444	0.384	0.632

Note: LOY = Customer loyalty; SAT = Customer satisfaction; CCI = Customer–company identification; ENV = Perceived environmental responsibility; SOC = Perceived social responsibility.

**Table 6:** Measurement model compositional invariance assessment.

	Original Correlation	Correlation Permutation Mean	Permutation *p*-Values
Perceived environmental responsibility	0.999	0.999	0.281
Perceived social responsibility	0.998	0.996	0.552
Customer–company identification	0.999	0.999	0.138
Customer satisfaction	1.000	1.000	0.373
Customer loyalty	1.000	1.000	0.125

Note: Permutation *p*-values are two tailed, based on 1000 permutations.

**Table 7:** Generation X vs. Generation Y multigroup analysis.

**Direct Effects**							
	**Gen X Path Coef.**	**Gen X** ***p* Values**	**Gen Y** **Path Coef.**	**Gen Y** ***p* Values**	**Gen X–Gen Y**	**Permu-Tation** ***p*-Values**	**PLS-MGA** ***p*-Values**
ENV → CCI	0.285 ***	0.000	0.312 ***	0.000	−0.027	0.700	0.697
ENV → SAT	0.072	0.168	0.221 ***	0.000	−0.149 **	0.028	0.033
ENV → LOY	0.003	0.917	−0.019	0.554	0.022	0.639	0.625
SOC → CCI	0.125 **	0.018	0.177 ***	0.000	−0.052	0.430	0.456
SOC → SAT	0.301 ***	0.000	0.134 **	0.010	0.168 **	0.021	0.019
SOC → LOY	0.065 **	0.049	0.083 ***	0.009	−0.018	0.694	0.691
CCI → SAT	0.302 ***	0.000	0.205 ***	0.000	0.097	0.117	0.103
CCI → LOY	0.122 ***	0.000	0.055 **	0.037	0.066	0.104	0.112
SAT → LOY	0.702 ***	0.000	0.758 ***	0.000	−0.056	0.175	0.177
**Indirect Effects**							
	**Gen X** **Effect**	**Gen X** ***p* Values**	**Gen Y** **Effect**	**Gen Y** ***p* Values**	**Gen X–Gen Y**	**Permu-Tation** ***p*-Values**	**PLS-MGA** ***p*-Values**
ENV → SAT	0.086 ***	0.000	0.064 ***	0.000	0.022	0.354	0.383
ENV → LOY	0.146 ***	0.000	0.233 ***	0.000	−0.088 *	0.089	0.098
SOC → SAT	0.038 **	0.029	0.036 ***	0.002	0.001	0.925	0.953
SOC → LOY	0.253 ***	0.000	0.139 ***	0.001	0.115 **	0.038	0.039
CCI → LOY	0.212 ***	0.000	0.155 ***	0.000	0.056	0.222	0.209
**R Square**							
	**Gen X** **R Square**		**Gen X** **R Square**		**Gen X–Gen Y**	**Permu-Tation** ***p*-Values**	**PLS-MGA** ***p*-Values**
LOY	0.625		0.642		−0.017	0.717	0.715

Note: * significant at 10% level; ** significant at 5% level; *** significant at 1% level; PLS-SEM and PLS-MGA *p*-values are two tailed, based on bootstrapping with 5000 subsamples; permutation *p*-values are two tailed, based on 1000 permutations; acronyms: LOY = Customer loyalty; SAT = Customer satisfaction; CCI = Customer–company identification; ENV = Perceived environmental responsibility; SOC = Perceived social responsibility.

**Table 8:** Generation X vs. Generation Y model predictive power assessment.

	Generation X				Generation Y			
	Q^2^_Predict_PLS_	RMSE_PLS_	RMSE_LM_	RMSE_PLS_ < RMSE_LM_	Q^2^_Predict_PLS_	RMSE_PLS_	RMSE_LM_	RMSE_PLS_ < RMSE_LM_
LOY1	0.128	1.394	1.401	Yes	0.112	1.508	1.519	Yes
LOY2	0.136	1.364	1.381	Yes	0.125	1.359	1.368	Yes
LOY3	0.122	1.322	1.331	Yes	0.080	1.342	1.350	Yes

Note: PLSpredict procedure with 10 folds and 10 repetitions; PLS = prediction using PLS-SEM; LM = prediction using a linear model; RMSE = root mean squared error.
